# Supplementary material for: Machine Learning Accelerates Crystallization for Structure Determination
Source: Angew Chem Int Ed Engl. 2026 May 4;65(25):e1218503. doi: 10.1002/anie.1218503 (PMC13266958; doi:10.1002/anie.1218503)

checkCIF/PLATON report

Structure factors have been supplied for datablock(s) lcz087

THIS REPORT IS FOR GUIDANCE ONLY. IF USED AS PART OF A REVIEW PROCEDURE FOR PUBLICATION, IT SHOULD NOT REPLACE THE EXPERTISE OF AN EXPERIENCED CRYSTALLOGRAPHIC REFEREE.

No syntax errors found. CIF dictionary Interpreting this report

Datablock: lcz087

Bond precision:	C-C = 0.0292 A		Wavelength=1.54184
Cell:	a=12.7861(2)	b=30.3212(4)	c=12.9477(2)
	alpha=90	beta=117.261(2)	gamma=90
Temperature:	100 K		

	Calculated	Reported
Volume	4462.15(14)	4462.15(13)
Space group	P 21	P 1 21 1
Hall group	P 2yb	P 2yb
Moiety formula	C27 H12 Ag3 F18 N7 S, 2(C15 H3 Ag3 F18 N6), C12 H9 N S	3(C15 H3 Ag3 F18 N6), 2(C12 H9 N S)
Sum formula	C69 H27 Ag9 F54 N20 S2	C69 H27 Ag9 F54 N20 S2
Mr	3197.06	3197.05
Dx,g cm-3	2.379	2.379
Z	2	2
Mu (mm-1)	17.494	17.494
F000	3044.0	3044.0
F000'	3060.09	
h,k,lmax	16,38,16	16,36,16
Nref	19023[9696]	13408
Tmin,Tmax	0.138,0.103	0.228,1.000
Tmin'	0.038	

Correction method= # Reported T Limits: Tmin=0.228 Tmax=1.000
AbsCorr = MULTI-SCAN

Data completeness= 1.38/0.70 Theta(max)= 77.748

R(reflections)= 0.0581(12427) wR2(reflections)= 0.1349(13408)

S = 1.095 Npar= 1388

The following ALERTS were generated. Each ALERT has the format
test-name_ALERT_alert-type_alert-level.
Click on the hyperlinks for more details of the test.

Alert level B

```
PLAT342_ALERT_3_B Low Bond Precision on  C-C Bonds ..............    0.02917 Ang.
```

Author Response: Disordered structure.

```
PLAT915_ALERT_3_B No Flack x Check Done: Low Friedel Pair Coverage         44 %
```

Author Response: Due to insufficient data. The compound crystallizes in the chiral space group, but no chiral ligand is used, and the complex is no longer chiral.

Alert level C

```
PLAT042_ALERT_1_C Calc. and Reported MoietyFormula Strings  Differ     Please Check
              Calc: C27 H12 Ag3 F18 N7 S, 2(C15 H3 Ag3 F18 N6), C1
                    2 H9 N S
              Rep.: 3(C15 H3 Ag3 F18 N6), 2(C12 H9 N S)
PLAT090_ALERT_3_C Poor Data / Parameter Ratio (Zmax > 18) ........       6.70 Note
PLAT213_ALERT_2_C Atom F00L            has ADP max/min Ratio .....        3.3 prolat
PLAT213_ALERT_2_C Atom F00Z            has ADP max/min Ratio .....        3.3 prolat
PLAT213_ALERT_2_C Atom F025            has ADP max/min Ratio .....        3.5 prolat
PLAT213_ALERT_2_C Atom F02F            has ADP max/min Ratio .....        3.6 prolat
PLAT213_ALERT_2_C Atom F02H            has ADP max/min Ratio .....        3.6 prolat
PLAT213_ALERT_2_C Atom F02O            has ADP max/min Ratio .....        3.1 prolat
PLAT234_ALERT_4_C Large Hirshfeld Difference F014     --C02U     .       0.18 Ang.
PLAT234_ALERT_4_C Large Hirshfeld Difference F02R     --C049     .       0.20 Ang.
PLAT234_ALERT_4_C Large Hirshfeld Difference F03D     --C02U     .       0.18 Ang.
PLAT234_ALERT_4_C Large Hirshfeld Difference F03N     --C042     .       0.22 Ang.
PLAT234_ALERT_4_C Large Hirshfeld Difference F01G     --C03T     .       0.19 Ang.
PLAT250_ALERT_2_C Large U3/U1 Ratio for <U(i,j)> Tensor(Resd    2)        2.3 Note
PLAT250_ALERT_2_C Large U3/U1 Ratio for <U(i,j)> Tensor(Resd    4)        2.1 Note
PLAT906_ALERT_3_C Large K Value in the Analysis of Variance ......      3.072 Check
PLAT911_ALERT_3_C Missing FCF Refl Between Thmin & STh/L=    0.600         48 Report
                1  0  0,   2 32  0,   3 32  0,   5 32  0,   0 36  0,   1 36  0,
               -1  0  1,   0  0  1,  -1  1  1,  -7 31  1,  -5 31  1,   6 31  1,
               -4 33  1,   2 33  1,   1 34  1,   4 34  1,   3 35  1,  -2 36  1,
               -1 36  1,   0 36  1,  -7 30  2,  -5 31  2,  -4 32  2,   2 34  2,
                0 35  2,  -1 36  2,   9 21  3,  -7 29  3,  -6 29  3,  -5 29  3,
                  (   18 More Missing: see the .ckf listing file)
```

```
PLAT971_ALERT_2_C Check Calcd Resid. Dens.  0.92Ang From Ag01          2.03 eA-3
PLAT971_ALERT_2_C Check Calcd Resid. Dens.  0.87Ang From F01G          1.81 eA-3
PLAT971_ALERT_2_C Check Calcd Resid. Dens.  1.19Ang From Ag03          1.81 eA-3
PLAT971_ALERT_2_C Check Calcd Resid. Dens.  0.97Ang From Ag02          1.78 eA-3
PLAT971_ALERT_2_C Check Calcd Resid. Dens.  0.98Ang From F043          1.76 eA-3
PLAT971_ALERT_2_C Check Calcd Resid. Dens.  1.04Ang From Ag07          1.76 eA-3
PLAT971_ALERT_2_C Check Calcd Resid. Dens.  1.21Ang From F043          1.71 eA-3
PLAT971_ALERT_2_C Check Calcd Resid. Dens.  1.01Ang From Ag01          1.69 eA-3
PLAT971_ALERT_2_C Check Calcd Resid. Dens.  1.01Ang From Ag05          1.69 eA-3
PLAT971_ALERT_2_C Check Calcd Resid. Dens.  0.95Ang From Ag05          1.61 eA-3
PLAT971_ALERT_2_C Check Calcd Resid. Dens.  0.96Ang From Ag08          1.51 eA-3
PLAT971_ALERT_2_C Check Calcd Resid. Dens.  0.89Ang From Ag04          1.51 eA-3
PLAT972_ALERT_2_C Check Calcd Resid. Dens.  0.77Ang From Ag02         -1.70 eA-3
PLAT972_ALERT_2_C Check Calcd Resid. Dens.  0.68Ang From Ag05         -1.54 eA-3
PLAT972_ALERT_2_C Check Calcd Resid. Dens.  0.65Ang From Ag02         -1.52 eA-3
PLAT977_ALERT_2_C Check Negative Difference Density on H02Q      .      -0.49 eA-3
PLAT977_ALERT_2_C Check Negative Difference Density on H03U      .      -0.71 eA-3
```

Alert level G

```
PLAT003_ALERT_2_G Number of Uiso or U(i,j)  Restrained non-H-Atoms         33 Report
PLAT007_ALERT_5_G Number of Unrefined Donor-H Atoms .............          2 Report
              H02E  H02Q
PLAT083_ALERT_2_G SHELXL Second Parameter in WGHT  Unusually Large      87.37 Why ?
PLAT177_ALERT_4_G The CIF-Embedded .res File Contains DELU Records          1 Report
PLAT186_ALERT_4_G The CIF-Embedded .res File Contains ISOR Records          3 Report
PLAT192_ALERT_3_G A Non-default DELU Restraint Value for SecondPar     0.0200 Report
PLAT242_ALERT_2_G Low    'MainMol' Ueq as Compared to Neighbors of       C02P Check
PLAT242_ALERT_2_G Low    'MainMol' Ueq as Compared to Neighbors of       C02U Check
PLAT242_ALERT_2_G Low    'MainMol' Ueq as Compared to Neighbors of       C035 Check
PLAT242_ALERT_2_G Low    'MainMol' Ueq as Compared to Neighbors of       C03Q Check
PLAT242_ALERT_2_G Low    'MainMol' Ueq as Compared to Neighbors of       C03R Check
PLAT242_ALERT_2_G Low    'MainMol' Ueq as Compared to Neighbors of       C049 Check
PLAT242_ALERT_2_G Low    'MainMol' Ueq as Compared to Neighbors of       C02C Check
PLAT242_ALERT_2_G Low    'MainMol' Ueq as Compared to Neighbors of       C037 Check
PLAT242_ALERT_2_G Low    'MainMol' Ueq as Compared to Neighbors of       C03C Check
PLAT242_ALERT_2_G Low    'MainMol' Ueq as Compared to Neighbors of       C03O Check
PLAT242_ALERT_2_G Low    'MainMol' Ueq as Compared to Neighbors of       C041 Check
PLAT242_ALERT_2_G Low    'MainMol' Ueq as Compared to Neighbors of       C042 Check
PLAT242_ALERT_2_G Low    'MainMol' Ueq as Compared to Neighbors of       C027 Check
PLAT242_ALERT_2_G Low    'MainMol' Ueq as Compared to Neighbors of       C02Y Check
PLAT242_ALERT_2_G Low    'MainMol' Ueq as Compared to Neighbors of       C03B Check
PLAT242_ALERT_2_G Low    'MainMol' Ueq as Compared to Neighbors of       C03P Check
PLAT242_ALERT_2_G Low    'MainMol' Ueq as Compared to Neighbors of       C03T Check
PLAT242_ALERT_2_G Low    'MainMol' Ueq as Compared to Neighbors of       C047 Check
PLAT434_ALERT_2_G Short Inter HL..HL Contact F00C     ..F044     .       2.79 Ang.
                                                   x,y,1+z  =     1_556 Check
PLAT434_ALERT_2_G Short Inter HL..HL Contact F00L     ..F011     .       2.81 Ang.
                                                   1+x,y,z  =     1_655 Check
PLAT434_ALERT_2_G Short Inter HL..HL Contact F00L     ..F025     .       2.82 Ang.
                                                 1+x,y,1+z  =     1_656 Check
PLAT434_ALERT_2_G Short Inter HL..HL Contact F012     ..F022     .       2.82 Ang.
                                                     x,y,z  =     1_555 Check
PLAT434_ALERT_2_G Short Inter HL..HL Contact F01G     ..F03E     .       2.79 Ang.
                                                 1+x,y,1+z  =     1_656 Check
PLAT434_ALERT_2_G Short Inter HL..HL Contact F01J     ..F01N     .       2.83 Ang.
                                                  x,y,-1+z  =     1_554 Check
```

```
PLAT434_ALERT_2_G Short Inter HL..HL Contact F01L     ..F039     .       2.75 Ang.
                                                        x,y,z  =     1_555 Check
PLAT720_ALERT_4_G Number of Unusual/Non-Standard Labels .........        181 Note
              Ag01    Ag02    Ag03    Ag04    Ag05    Ag06    Ag07    Ag08
              Ag09    S00A    S00B    F00C    F00D    F00E    F00F    F00G
              N00H    F00I    N00J    N00K    F00L    N00M    F00N    F00O
              N00P    F00Q    N00R    F00S    N00T    F00U    N00V    N00W
              N00X    N00Y    F00Z    C010    H010    F011    F012    N013
              F014    F015    F016    N017    F018    C019    F01A    F01B
              C01C    C01D    F01E    C01F    H01F    F01G    C01H    C01I
              F01J    F01K    F01L    F01M    F01N    C01O    H01O    F01P
              C01Q    F01R    C01S    C01T    C01U    F01V    N01W    N01X
              F01Y    C01Z    H01Z    F020    F021    F022    C023    C024
              H024    F025    C026    C027    C028    H028    C029    H029
              N02A    N02B    C02C    N02D    N02E    H02E    F02F    C02G
              F02H    C02I    C02J    H02J    C02K    C02L    C02M    C02N
              F02O    C02P    N02Q    H02Q    F02R    C02S    C02T    C02U
              C02V    H02V    C02W    C02X    H02X    C02Y    C02Z    H02Z
              F030    F031    C032    H032    C033    H033    C034    C035
              C036    C037    C038    F039    C03A    H03A    C03B    C03C
              F03D    F03E    C03F    H03F    C03G    H03G    F03H    C03I
              H03I    F03J    F03K    C03L    H03L    F03M    F03N    C03O
              C03P    C03Q    C03R    C03S    H03S    C03T    C03U    H03U
              C03V    C03W    H03W    C03X    F03Y    C03Z    H03Z    C040
              C041    C042    F043    F044    C045    H045    C046    H046
              C047    C049    C04A    H04A    F04B
PLAT860_ALERT_3_G Number of Least-Squares Restraints ............        200 Note
PLAT912_ALERT_4_G Missing # of FCF Reflections Above STh/L=  0.600        316 Note
PLAT933_ALERT_2_G Number of HKL-OMIT Records in Embedded .res File         43 Note
               -9 29  2,  -8 24  4,  -8 31  1,  -7 22  5,  -7 27  4,  -7 29  3,
               -7 30  2,  -7 31  1,  -6 28  4,  -6 29  3,  -6 31  3,  -5 26  5,
               -5 27  4,  -5 28  4,  -5 29  3,  -5 29  4,  -5 30  3,  -5 31  1,
               -5 31  2,  -5 31  3,  -4-17  2,  -4 25  5,  -4 30  3,  -4 32  2,
               -4 33  1,   1 34  1,   2 32  0,   2 33  1,   2 34  2,   2 36  0,
                2 36  1,   3 32  0,   3 35  1,   3 35  3,   3 36  2,   4 34  1,
                4 34  2,   4 36  1,   5 32  0,   5 34  2,   6 31  3,   6 32  2,
                6 33  1,
PLAT941_ALERT_3_G Average HKL Measurement Multiplicity ..........        3.1 Low
PLAT951_ALERT_5_G Calculated (ThMax) and CIF-Reported Kmax Differ          2 Units
PLAT957_ALERT_1_G Calculated (ThMax) and Actual (FCF) Kmax Differ          2 Units
PLAT969_ALERT_5_G The 'Henn et al.' R-Factor-gap value ..........      3.010 Note
              Predicted wR2: Based on SigI**2  4.48 or SHELX Weight 12.32
PLAT978_ALERT_2_G Number C-C Bonds with Positive Residual Density.          0 Info
```

```
   0 ALERT level A = Most likely a serious problem - resolve or explain
   2 ALERT level B = A potentially serious problem, consider carefully
  34 ALERT level C = Check. Ensure it is not caused by an omission or oversight
  40 ALERT level G = General information/check it is not something unexpected

   2 ALERT type 1 CIF construction/syntax error, inconsistent or missing data
  54 ALERT type 2 Indicator that the structure model may be wrong or deficient
   8 ALERT type 3 Indicator that the structure quality may be low
   9 ALERT type 4 Improvement, methodology, query or suggestion
   3 ALERT type 5 Informative message, check
```

Validation response form

Please find below a validation response form (VRF) that can be filled in and pasted into your CIF.

```
# start Validation Reply Form
_vrf_PLAT042_lcz087
;
PROBLEM: Calc. and Reported MoietyFormula Strings  Differ     Please Check
RESPONSE: ...
;
_vrf_PLAT090_lcz087
;
PROBLEM: Poor Data / Parameter Ratio (Zmax > 18) ........       6.70 Note
RESPONSE: ...
;
_vrf_PLAT213_lcz087
;
PROBLEM: Atom F00L            has ADP max/min Ratio .....        3.3 prolat
RESPONSE: ...
;
_vrf_PLAT234_lcz087
;
PROBLEM: Large Hirshfeld Difference F014     --C02U     .       0.18 Ang.
RESPONSE: ...
;
_vrf_PLAT250_lcz087
;
PROBLEM: Large U3/U1 Ratio for <U(i,j)> Tensor(Resd    2)        2.3 Note
RESPONSE: ...
;
_vrf_PLAT906_lcz087
;
PROBLEM: Large K Value in the Analysis of Variance ......      3.072 Check
RESPONSE: ...
;
_vrf_PLAT911_lcz087
;
PROBLEM: Missing FCF Refl Between Thmin & STh/L=    0.600         48 Report
RESPONSE: ...
;
_vrf_PLAT971_lcz087
;
PROBLEM: Check Calcd Resid. Dens.  0.92Ang From Ag01            2.03 eA-3
RESPONSE: ...
;
_vrf_PLAT972_lcz087
;
PROBLEM: Check Calcd Resid. Dens.  0.77Ang From Ag02           -1.70 eA-3
RESPONSE: ...
;
_vrf_PLAT977_lcz087
;
PROBLEM: Check Negative Difference Density on H02Q      .      -0.49 eA-3
RESPONSE: ...
;
# end Validation Reply Form
```

It is advisable to attempt to resolve as many as possible of the alerts in all categories. Often the minor alerts point to easily fixed oversights, errors and omissions in your CIF or refinement strategy, so attention to these fine details can be worthwhile. It is up to the individual to critically assess their own results and, if necessary, seek expert advice.

PLATON version of 17/09/2025; check.def file version of 17/09/2025

---

duplicate check

No duplication found

Datablock lcz087 - ellipsoid plot

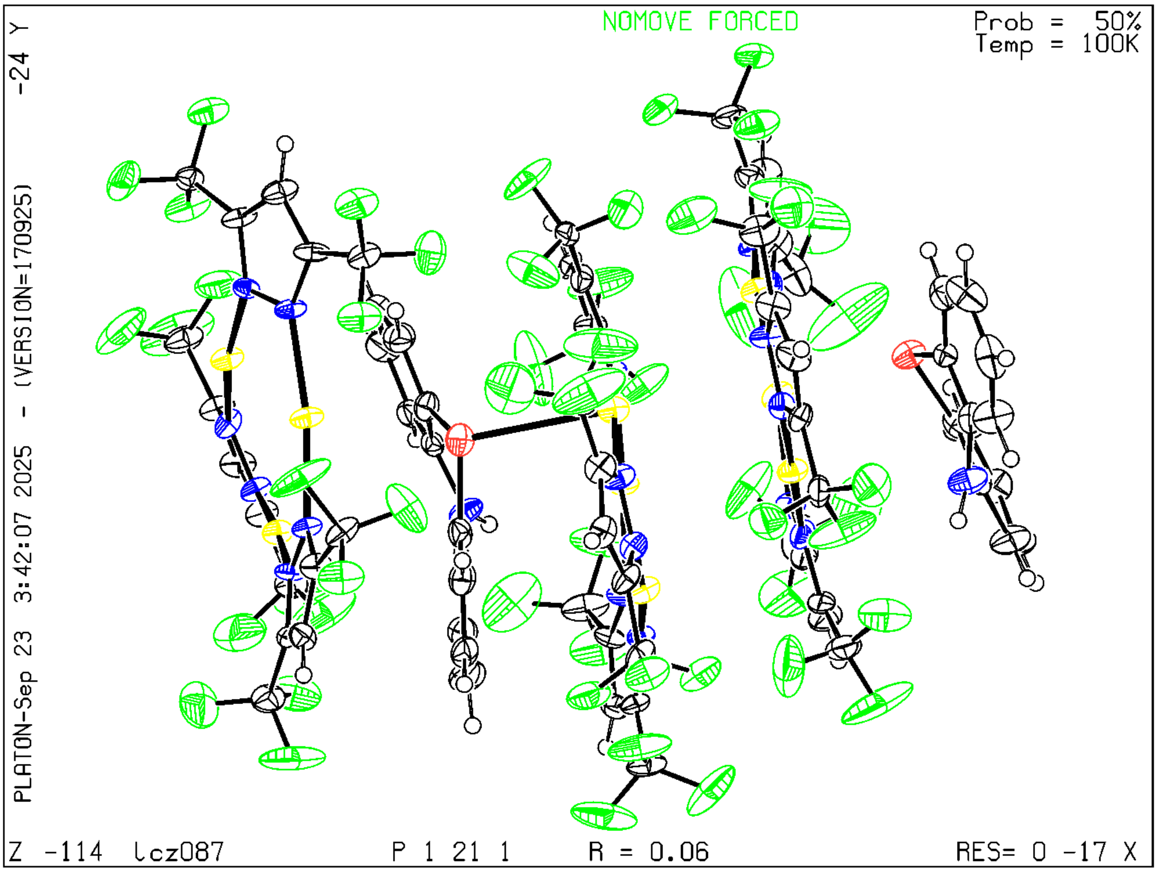

Supplement: Supplementary file 2 — Supporting File 2: anie72462‐sup‐0002‐DataFile.zip. [file ANIE-65-e1218503-s002.zip › supplementary_materials_crystallographic_data submit.zip!/cif_and_checkcif(1-114)/Ag3Pz3_87'/Ag3Pz3_87'_checkcif.pdf]
